# Diagnostic Challenge of Nodal Nevi Mimicking Metastatic Melanoma in Axillary Lymph Nodes Following Neoadjuvant Therapy for Breast Cancer: A Case Report

**DOI:** 10.7759/cureus.90745

**Published:** 2025-08-22

**Authors:** Ahlam Albloshi, Salama Samih, Salem Alowami, Peter Lovrics, Pooja Vasudev

**Affiliations:** 1 Pathology and Molecular Medicine, McMaster University, St Joseph’s Healthcare, Hamilton, CAN; 2 Surgery, McMaster University, St Joseph’s Healthcare, Hamilton, CAN

**Keywords:** breast cancer, immunohistochemical markers, ki-67, metastatic melanoma, nodal nevi, prame

## Abstract

Nodal nevi are benign melanocytic proliferations within lymph nodes that can closely mimic metastatic melanoma, posing a significant diagnostic challenge, particularly in breast cancer patients undergoing lymph node dissection after neoadjuvant chemotherapy. Accurate differentiation between nodal nevi and true melanoma metastases is essential to avoid misdiagnosis and overtreatment. Immunohistochemical (IHC) markers such as preferentially expressed antigen in melanoma (PRAME), p16, human melanoma black-45 (HMB-45), and Ki-67 are critical tools for diagnostic clarification. We present a diagnostically challenging case of multiple infiltrative nodal nevi in a 59-year-old female with triple-negative invasive ductal carcinoma, no special type, of the breast. The patient had a prior history of dysplastic nevus on the upper trunk and presented with a 1.5 cm palpable mass in the left breast and a 5 cm left axillary mass. Following neoadjuvant chemotherapy, both lesions demonstrated a clinical reduction in size. She subsequently underwent a partial mastectomy and axillary lymph node dissection. Histologic examination revealed no residual invasive carcinoma in the breast. However, four axillary lymph nodes contained atypical melanocytic-appearing cells in the subcapsular sinuses with extension into the nodal parenchyma, raising the differential diagnosis of residual carcinoma versus metastatic melanoma. Initial IHC showed these atypical cells to be melanocytic in origin (SOX10 and melanoma cocktail positive; AE1/AE3 negative). While initial interpretation favored metastatic melanoma, further IHC workup demonstrated low proliferative activity (Ki-67 <1%), diffuse p16 positivity, and negativity for both HMB-45 and PRAME. These findings, along with dermatopathology consultation, supported a diagnosis of multiple nodal nevi rather than melanoma. This case underscores the diagnostic pitfall posed by infiltrative nodal nevi, particularly when they mimic melanoma in the setting of breast cancer. It highlights the importance of comprehensive immunohistochemical panels, including PRAME, p16, HMB-45, and Ki-67, and the value of second opinions and dermatopathology consultation in avoiding diagnostic error.

## Introduction

Nodal nevi are benign melanocytic proliferations within lymph nodes that can histologically mimic metastatic melanoma, particularly in the setting of sentinel lymph node biopsies or axillary lymph node dissections performed for patients with breast carcinoma or cutaneous melanoma [1-3]. This diagnostic dilemma is especially relevant in breast cancer patients undergoing sentinel lymph node biopsy or axillary dissection following neoadjuvant therapy, where the presence of nodal nevi can complicate interpretation. Accurate distinction between nodal nevi and true melanoma metastasis is critical to prevent misdiagnosis and its potential consequences, including unnecessary surgical intervention, inappropriate adjuvant therapy, and psychological distress [1-3].

In the current report, we describe a 59-year-old woman with triple-negative breast carcinoma who, following neoadjuvant chemotherapy, was found to have multifocal melanocytic proliferations in axillary lymph nodes. The unusual infiltrative pattern initially raised concern for metastatic melanoma, prompting further immunohistochemical and expert dermatopathology evaluation.

Histopathologic evaluation remains the diagnostic cornerstone; however, reliance on morphology alone may be insufficient due to overlapping features such as melanocytic nesting, pigmentation, bland cytology, absence of atypia and mitoses, and involvement of the lymph node parenchyma [1-5]. While nodal nevus cells are most commonly located in the capsule or trabeculae, intraparenchymal nevus cells are exceedingly rare and may pose a greater diagnostic challenge [6].

Immunohistochemistry (IHC) provides crucial ancillary information. Preferentially expressed antigen in melanoma (PRAME) is highly expressed in malignant melanoma but not in benign melanocytic proliferations [1]. Similarly, human melanoma black-45 (HMB-45) expression is typically present in about half of melanoma cases but is often lost in mature nevi [4-6]. Ki-67, a proliferation marker, shows elevated activity in melanoma but is generally low or absent in nodal nevi [2,5]. P16, although variably expressed, may provide useful supportive data when interpreted alongside other markers [1,3]. Additional ancillary markers such as Melan-A and SOX10 are sensitive for melanocytic differentiation and can help confirm melanocytic lineage; however, they do not distinguish between benign melanocytic proliferations (e.g., nodal nevi) and malignant melanoma [7].

We report a diagnostically challenging case of multiple nodal nevi identified in axillary lymph nodes during breast cancer surgery. Initial histomorphologic and immunophenotypic features raised suspicion for metastatic melanoma; however, further IHC analysis, expert dermatopathology consultation, and clinicopathologic correlation ultimately confirmed the benign nature of these melanocytic proliferations. This case is clinically significant given the rarity of multifocal and infiltrative intraparenchymal nodal nevi, which can closely mimic metastatic melanoma and present a major diagnostic pitfall in the setting of breast cancer.

## Case presentation

Clinical history

A 59-year-old woman with a remote history of a dysplastic nevus on the upper trunk presented with a 1.5 cm palpable mass in the left breast and a 5.0 cm fixed mass in the left axilla. She had no personal or family history of breast or ovarian cancer, and *BRCA* genetic testing was negative. She had no history of melanoma.

Imaging and diagnosis

**Figure 1:**
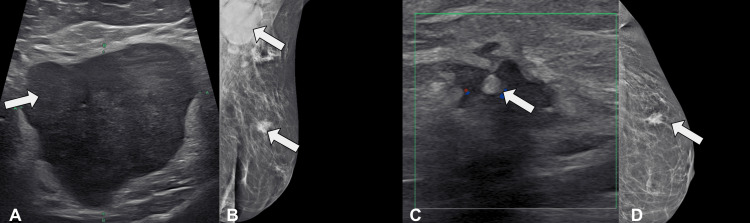
Initial diagnostic imaging of the left breast and axilla. (A) Axillary ultrasound showing an enlarged hypoechoic lymph node with irregular borders (arrowhead). (B) Mammogram showing a spiculated breast mass and enlarged axillary lymph node (arrowheads). (C) Breast ultrasound showing an irregular hypoechoic mass with posterior acoustic shadowing (arrowhead). (D) Craniocaudal mammogram showing a spiculated mass in the upper outer quadrant (arrowhead).

**Figure 2:**
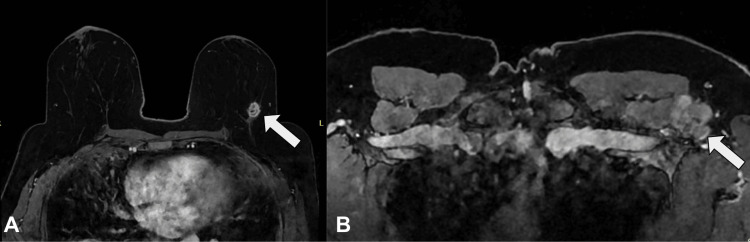
Pre-neoadjuvant breast and axilla MRI. (A) Breast MRI showing an avidly enhancing spiculated mass in the upper outer quadrant at the 2 o’clock position (arrowhead). (B) Axillary MRI showing an irregular mass with invasion of the pectoralis minor muscle (arrowhead).

Diagnostic mammography and targeted ultrasound of the left breast and axilla revealed a 1.6 × 1.5 × 1.7 cm irregular mass in the upper outer quadrant at the 2 o’clock position, 5 cm from the nipple, and enlarged axillary lymph nodes measuring up to 5.1 × 4.6 × 4.2 cm (Figure 1). Breast MRI confirmed an enhancing mass with mildly spiculated margins in the left breast and an irregular axillary mass invading the pectoralis minor muscle (Figure 2). The right breast and axilla were unremarkable.

Ultrasound-guided core needle biopsy of the breast lesion confirmed invasive ductal carcinoma, no special type, Nottingham grade 2, with a triple-negative immunophenotype (estrogen receptor-negative, progesterone receptor-negative, human epidermal growth factor receptor 2-negative). Biopsy of the axillary lymph node revealed metastatic carcinoma consistent with a breast primary. Staging CT and bone scans showed no evidence of distant metastasis.

Treatment course

The patient underwent neoadjuvant systemic chemotherapy consisting of five months of dose-dense doxorubicin and cyclophosphamide, followed by paclitaxel. In the sixth month, additional cycles of chemotherapy were administered in combination with pembrolizumab. This was followed by adjuvant pembrolizumab (4 mg/kg every six weeks) for three months. The treatment course was well tolerated and uneventful.

**Figure 3:**
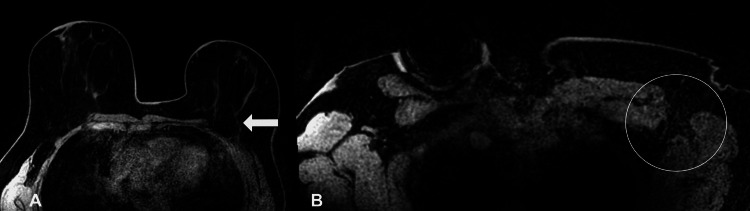
Post-treatment MRI of the breast and axilla. (A) Left breast MRI showing linear non-mass enhancement at the prior tumor site (arrowhead), consistent with treatment response and no residual mass. (B) Left axilla MRI showing marked reduction in lymphadenopathy (open circle), indicating favorable response to therapy.

Post-treatment clinical examination revealed no palpable masses in the left breast or axilla. MRI demonstrated only linear non-mass enhancement in the breast and a significant reduction in axillary lymphadenopathy (Figure 3). Given the favorable response, the patient underwent a left partial mastectomy with seed localization and axillary lymph node dissection. A sentinel lymph node biopsy was not performed due to confirmed nodal involvement before treatment. A total of 18 lymph nodes were dissected. Gross examination of the breast specimen showed treatment-related fibrosis without residual tumor. Histologic examination confirmed a complete pathologic response (pCR) in the breast.

Pathologic findings

**Figure 4:**
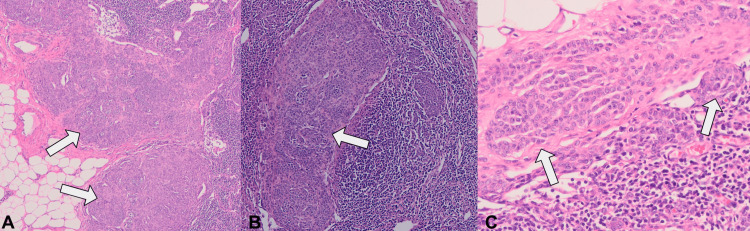
Histopathology of axillary lymph nodes with melanocytic proliferation. (A) Low-power view showing melanocytic nests infiltrating the lymph node parenchyma (arrowheads) (hematoxylin and eosin (H&E), ×20). (B) Intermediate-power view highlighting melanocytic nests within the parenchyma (arrowhead) (H&E, ×100). (C) High-power view demonstrating intracapsular and subcapsular melanocytic nests (arrowheads) (H&E, ×200).

Four axillary lymph nodes demonstrated well-demarcated melanocytic proliferations within the subcapsular sinus extending into the parenchyma, the largest deposit measuring 8 mm. Histologic sections showed monomorphic melanocytic cells arranged in nests and single cells infiltrating intracapsular and subcapsular trabeculae. The cells exhibited mild nuclear atypia, pale cytoplasm, and inconspicuous nucleoli; rare mitotic figures were identified (Figure 4). The morphology raised concern for metastatic melanoma or, less likely, metastatic breast carcinoma with unusual post-therapy morphology.

**Figure 5:**
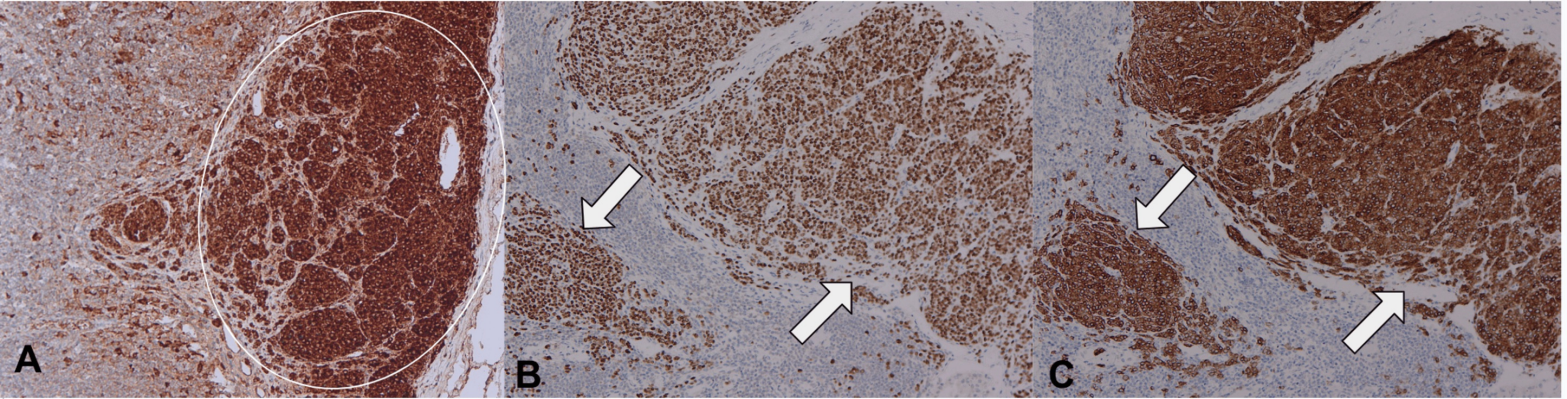
Immunohistochemistry of melanocytic proliferation in axillary lymph node. (A) Strong cytoplasmic positivity for S100 in melanocytic cells (open circle) (S100, ×100). (B) Strong nuclear positivity for SOX10 in melanocytic cells (arrowheads) (SOX10, ×100). (C) Diffuse cytoplasmic positivity for melanoma cocktail in melanocytic cells (arrowheads) (Melanoma cocktail, ×100).

**Figure 6:**
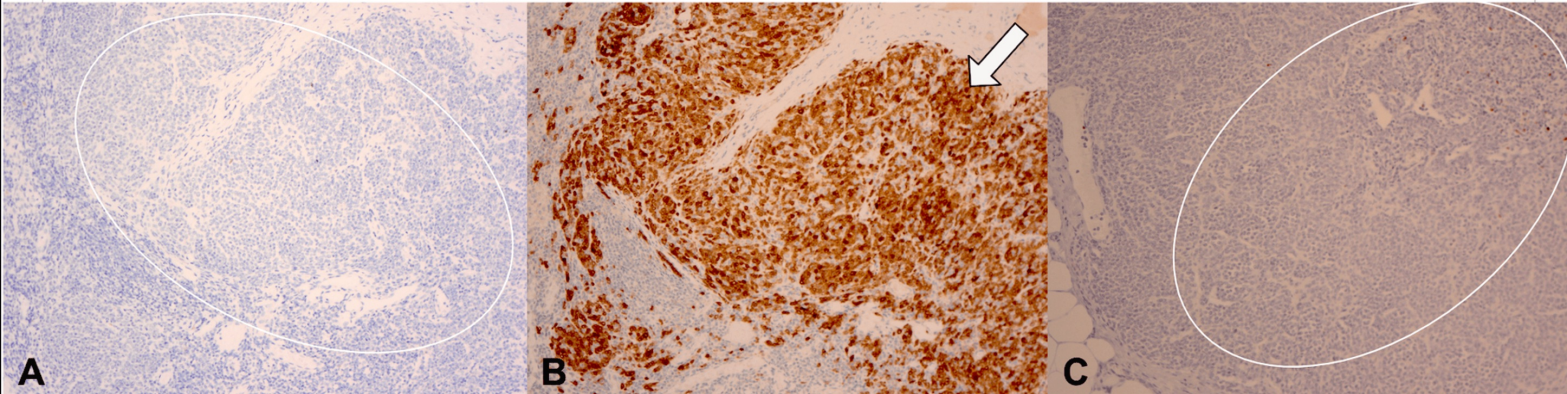
Additional immunohistochemistry of melanocytic proliferation in axillary lymph node. (A) Negative HMB-45 staining in melanocytic cells (open circle) (HMB-45, ×100). (B) Nuclear and cytoplasmic positivity for p16 in melanocytic cells (arrowhead) (p16, ×100). (C) Very low proliferative activity (<1%) by Ki-67 (open circle) (Ki-67, ×100).

Immunohistochemical studies demonstrated strong positivity for S100, SOX10, and melanoma cocktail (MART-1/Melan-A, HMB-45, and tyrosinase), with negativity for AE1/AE3, CAM5.2, CK7, and HMB-45. P16 expression was retained, and the Ki-67 index was <1% (Figure 5). A second panel, including PRAME, performed by a consulting dermatopathologist, showed complete negativity (Figure 6).

Taken together, the immunoprofile (PRAME negativity, retained p16, low mitotic index, HMB-45 loss, and absence of epithelial markers), combined with the morphologic features and clinical history, supported a diagnosis of benign nodal nevi. This interpretation was further reinforced by the patient’s history of a dysplastic nevus and the absence of clinical or radiologic evidence of melanoma. Despite the unusual multifocal and infiltrative pattern, integration of clinical, radiologic, histologic, and immunophenotypic data, along with dermatopathology consultation, allowed for confident exclusion of both metastatic melanoma and recurrent breast carcinoma. Table 1 presents a summary of the diagnostic findings. Table 2 compares the IHC profile and the differential diagnosis.

**Table 1:** Summary of diagnostic findings. This table summarizes the key clinical, imaging, and histopathologic findings of the case. MRI = magnetic resonance imaging; pCR = pathologic complete response; BRCA = germline BRCA mutation status; triple-negative = estrogen receptor-negative, progesterone receptor-negative, human epidermal growth factor receptor 2-negative breast carcinoma

Category	Findings
Clinical	A 59-year-old woman; palpable left breast mass (1.5 cm) and fixed left axillary mass (5.0 cm); a history of dysplastic nevus; no history of melanoma; BRCA negative; breast carcinoma was triple-negative (ER-, PR-, HER2-)
Imaging	Mammography/Ultrasound: an irregular breast mass (1.6 cm) and enlarged axillary nodes (5.1 cm). MRI: spiculated enhancing breast mass and axillary mass invading pectoralis minor. Post-treatment MRI: complete response in breast; reduced axillary disease
Histopathology	Breast: treatment-related fibrosis, no residual carcinoma (pCR). Lymph nodes: melanocytic proliferations (up to 8 mm) with mild atypia, rare mitoses, no extranodal extension; multifocal and infiltrative pattern mimicking metastatic melanoma. Metastatic invasive ductal carcinoma was considered in the differential

**Table 2:** Comparative immunohistochemical profile and differential diagnosis. This table summarizes the immunohistochemical findings of the case in comparison with major differential diagnoses. IHC = immunohistochemistry; IDC = invasive ductal carcinoma; PRAME = preferentially expressed antigen in melanoma; Ki-67 = proliferation index; AE1/AE3, CAM5.2, CK7 = epithelial markers

Marker	Findings in this case	Expected in metastatic melanoma	Expected in metastatic IDC	Interpretation
S100	Positive	Positive	Negative	Supports melanocytic lineage, not epithelial
SOX10	Positive	Positive	Negative	Confirms melanocytic differentiation
Melanoma cocktail (MART-1/Melan-A, HMB-45, tyrosinase)	Positive overall, but HMB-45 negative on individual stain	Usually diffusely positive (including HMB-45)	Negative	HMB-45 loss favors nodal nevus rather than melanoma
AE1/AE3, CAM5.2, CK7	Negative	Negative	Positive	Excludes metastatic breast carcinoma (IDC)
p16	Retained (positive)	Often lost in melanoma	Variable	Retention supports benign nevus over melanoma
PRAME	Negative	Positive in most melanomas	Negative	Negativity strongly argues against melanoma
Ki-67	<1% (low proliferation)	High (>10–20%) in melanoma	Variable, but higher than nevus	Very low index supports benign nevus

## Discussion

Nodal nevi are benign melanocytic proliferations within lymph nodes, most often identified incidentally during sentinel lymph node biopsy or lymph node dissection for malignancies such as cutaneous melanoma and breast carcinoma [1-3,7]. Although biologically benign, their histologic appearance, particularly when extending into the nodal parenchyma, can closely mimic metastatic melanoma, creating a significant diagnostic challenge. Misinterpretation may lead to unnecessary surgical procedures, inappropriate systemic therapy, and considerable psychological distress for patients [1,7-10].

In the present case, the multifocal and infiltrative growth pattern of melanocytic cells within axillary lymph nodes raised concern for metastatic melanoma. However, several morphologic features favored a benign process, including mild cytologic atypia, rare mitotic figures, absence of extranodal extension, and a very low proliferative index [2,5]. Nevertheless, morphology alone is often insufficient to distinguish between nodal nevi and melanoma [1,3].

IHC provides crucial ancillary information. PRAME, a melanoma-associated antigen, is a highly sensitive and specific marker for melanoma and is typically negative in benign nevi [1,3]. In our case, PRAME negativity strongly favored a benign diagnosis, and this finding was critical in excluding metastatic melanoma. Similarly, retained p16 expression and a Ki-67 labeling index of less than 1% further supported a benign process [1-3,5]. While SOX10 and S100 confirmed melanocytic origin, they do not distinguish benign from malignant melanocytic proliferations [7].

HMB-45, which highlights immature melanocytes, is usually expressed in melanomas but tends to be lost in maturing nevi, including nodal nevi [4-6,11]. Its absence in this case was consistent with a benign proliferation, providing further diagnostic reassurance.

Despite these helpful markers, no single IHC stain or standardized panel can definitively differentiate nodal nevi from melanoma in every instance [12]. Diagnostic certainty requires integration of morphologic, immunophenotypic, and clinical findings, often in consultation with dermatopathology experts [12]. In our patient, the combination of benign morphologic features, PRAME negativity, HMB-45 loss, preserved p16 expression, and low proliferative index, together with a history of dysplastic nevus and no clinical or radiologic evidence of melanoma, supported the final diagnosis of nodal nevi. Dermatopathology review confirmed this interpretation and excluded metastatic melanoma.

Nodal nevi are most commonly reported in sentinel lymph node biopsies performed for cutaneous melanoma, where their subcapsular location can mimic metastatic deposits [13]. Reports of nodal nevi in the setting of breast carcinoma are exceedingly rare, with only a few isolated case reports described [14,15]. Unlike the predominantly subcapsular distribution documented in melanoma-related cases, our patient demonstrated a multifocal and infiltrative pattern, posing a greater diagnostic challenge. Although this patient had received neoadjuvant chemotherapy, there is no evidence to suggest that treatment-related effects contribute to nevus proliferation within lymph nodes; thus, this finding is most consistent with a benign incidental phenomenon. This underscores the importance of integrating clinical, morphologic, and immunophenotypic data, in close collaboration with dermatopathology expertise, to avoid misdiagnosis and overtreatment.

## Conclusions

Distinguishing nodal nevi from metastatic melanoma remains one of the most nuanced and diagnostically demanding areas in surgical pathology. This case highlights the unique challenge posed by large, multifocal, and infiltrative nodal nevi, which can closely mimic melanoma both morphologically and immunohistochemically. Accurate distinction requires a systematic and integrated approach that combines histologic assessment, immunohistochemical profiling, including PRAME, p16, HMB-45, and Ki-67, and thorough clinical correlation. Although these advanced markers enhance diagnostic accuracy, they must always be interpreted in conjunction with classic morphologic parameters such as nuclear atypia, mitotic activity, and growth pattern. Notably, the absence of HMB-45 staining in mature nevi and its usual retention in melanoma provides a valuable diagnostic clue. However, melanocytic markers such as SOX10, S100, and MART-1, while confirming lineage, lack discriminatory power between benign and malignant lesions and therefore cannot be solely relied upon for this distinction. Robust clinical correlation and subspecialty consultation with dermatopathologists are often indispensable in resolving challenging melanocytic cases. Expert input ensures the appropriate application of IHC, fosters diagnostic confidence, and aligns findings with current diagnostic standards. Importantly, accurate recognition of nodal nevi has direct clinical implications, as misdiagnosis may lead to unnecessary axillary surgery, inappropriate systemic therapy, and significant psychological burden for patients. Looking ahead, further studies and consensus efforts are needed to establish standardized diagnostic protocols that enhance reproducibility, reduce diagnostic error, and, ultimately, improve patient outcomes.
